# Preventive Effects of an *Opuntia stricta* var. *dillenii* Extract on Lipid Metabolism in a High-Fat High-Fructose Diet-Induced Obesity Animal Model

**DOI:** 10.3390/nu17193178

**Published:** 2025-10-08

**Authors:** Iker Gómez-García, Alfredo Fernández-Quintela, Paula Oliver, Catalina Picó, M. Pilar Cano, María P. Portillo, Jenifer Trepiana

**Affiliations:** 1 Nutrition and Obesity Group, Department of Nutrition and Food Science, University of the Basque Country (UPV/EHU), 01006 Vitoria-Gasteiz, Spain; iker.gomez@ehu.eus (I.G.-G.); mariapuy.portillo@ehu.eus (M.P.P.); jenifer.trepiana@ehu.eus (J.T.); 2 Lucio Lascaray Research Institute, 01006 Vitoria-Gasteiz, Spain; 3 Bioaraba Health Research Institute, 01009 Vitoria-Gasteiz, Spain; 4 CIBERobn Physiopathology of Obesity and Nutrition, Institute of Health Carlos III, 28029 Madrid, Spain; paula.oliver@uib.es (P.O.); cati.pico@uib.es (C.P.); 5 Laboratory of Molecular Biology, Nutrition and Biotechnology (Nutrigenomics, Biomarkers and Risk Evaluation (NuBE) Group), University of the Balearic Islands, 07122 Palma, Spain; 6 Health Research Institute of the Balearic Islands (IdISBa), 07120 Palma, Spain; 7 Artificial Intelligence Research Institute of the Balearic Islands (IAIB), 07122 Palma, Spain; 8 Laboratory of Phytochemistry and Plant Food Functionality, Biotechnology and Food Microbiology Department, Institute of Food Science Research (CIAL) (CSIC-UAM), 28049 Madrid, Spain; mpilar.cano@csic.es

**Keywords:** *Opuntia stricta* var. *dillenii*, obesity, prickly pear, betalains, phenolic compounds, adipose tissue, high-fat high-fructose diet

## Abstract

**Background:** Due to the continuous global rise in obesity prevalence, foods rich in bioactive compounds are increasingly recognised for the management of several diseases. **Objective:** The present study aims to investigate whether an *Opuntia stricta* var. *dillenii* fruit peel extract, rich in betalains and phenolic compounds, is able to prevent obesity induced by a high-fat high-fructose diet in rats, along with the potential mechanisms of action underlying this effect. **Results:** The supplementation with *Opuntia stricta* var. *dillenii* extract obtained from the peel fruit partially prevents obesity development by attenuating HFHF-induced fat accumulation. This effect was observed predominantly in visceral adipose tissue, rather than in the subcutaneous depot. The obesity prevention was accompanied by the improvement of serum lipid profile. The mechanisms underlying the extract anti-obesity effect which were analysed in epididymal adipose tissue, involve preventing the rise in the availability of triglyceride synthesis substrates induced by high-fat high-fructose feeding, the inhibition of triglyceride assembly, and in the case of the high dose, increased lipolysis. **Conclusions:** According to these results, the peel wastes of *Opuntia stricta* var. *dillenii* fruit represent a promising natural source of bioactive compounds for obesity prevention. Nevertheless, these preclinical effects should be replicated in further studies in human beings.

## 1. Introduction

Obesity has been recognised as a disease with adverse effects requiring early detection and appropriate management [1]. The World Health Organization defines obesity as a body mass index (BMI) of 30 kg/m^2^ or greater. According to this source, 59% of European and 67.5% of American adults present overweight or obesity, and this prevalence continues to rise [2,3]. In the case of the child population, more than 390 million children and adolescents aged 5 to 19 years worldwide are affected by overweight [4]. Given the magnitude of the epidemic, the prevention and treatment of obesity have become key priorities for health systems.

With the aim of mitigating the prevalence of certain chronic diseases, the development of functional foods that improve health status is gaining attention within the scientific community. In this context, plants of the *Opuntia* genus, belonging to the *Cactaceae* family, produce prickly pear, a pear-shaped fruit exclusive to this genus, which are of considerable interest due to their high content of bioactive compounds, such as phenolic compounds, betalains and phytosterols, which may exert beneficial health effects [5,6,7]. To date, over 250 cactus species belonging to the *Opuntia* genus have been identified. They are mainly distributed throughout the Americas, with Mexico hosting the greatest species diversity, although they are also found in Africa, Asia, Oceania, and parts of the Mediterranean region [8].

**Figure 1:**
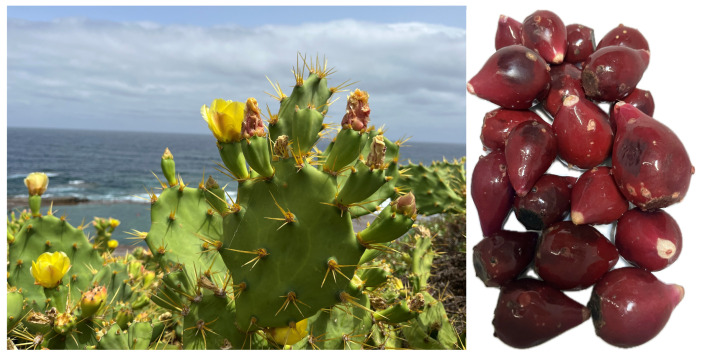
*Opuntia stricta* var. *dillenii* cactus and its fruit (prickly pear).

Among the various species of the *Opuntia* genus, *Opuntia ficus-indica* is the most widely known, commercialised and studied. To date, several investigations have been conducted on rodents fed diets supplemented with *Opuntia ficus-indica* extracts, obtained from different parts of the plant, demonstrating a reduction in body weight [9,10,11]. In addition, other species, including *Opuntia stricta* var. *dillenii* (Figure 1), which grows wild in the Canary Islands, have also shown beneficial effects on health [12,13]. Nevertheless, the potential of *Opuntia stricta* var. *dillenii* in obesity management remains unexamined. In fact, to the best of our knowledge, this is the first time that the *Opuntia stricta* var. *dillenii* peel extract has been studied in an in vivo obesity model.

The interest in utilising *Opuntia* extends beyond its health-related properties; environmental considerations have also been attributed to this genus. The Food and Agriculture Organization (FAO) has recognised *Opuntia* cacti as valuable in addressing current challenges such as prolonged droughts and desertification, as these plants can grow in arid regions and withstand harsh environmental conditions like rising atmospheric CO_2_ levels [14]. Their resilience not only contributes to preventing soil erosion and combating desertification but also supports their use as food and as a resource within sustainable agricultural and livestock systems.

In the case of *Opuntia stricta* var. *dillenii* fruits, the pulp is commercially used for various applications, particularly in the production of comfitures. However, this process generates a considerable amount of by-products, mainly the peel and seeds, which together represent approximately 37% to 67% of the total fruit weight [15]. These non-edible portions are generally discarded, despite containing valuable bioactive compounds, and their accumulation can pose environmental challenges due to the high volume of organic waste produced. In most industrial processes, only the pulp and juice are utilised, while the remaining components are largely underexploited, apart from their use as animal feed in certain countries [16]. Valorising these by-products through their incorporation into the development of nutraceuticals or functional foods not only improves the overall efficiency of fruit utilization but also supports the sustainable use of food resources. This approach aligns with the principles of the circular economy and directly contributes to several Sustainable Development Goals (SDGs), in particular, it could be part of the goals “Responsible consumption and production” (SDG 12) and “Climate action” (SDG 13), by reducing food waste, promoting resource efficiency, and mitigating environmental impact [17].

In this context, the present study aims to investigate whether an *Opuntia stricta* var. *dillenii* peel extract, rich in betalains and phenolic compounds, is able to prevent obesity induced by a high-fat high-fructose diet in rats, along with the potential mechanisms of action underlying this effect.

## 2. Materials and Methods

### 2.1. Reagents

Acetyl CoA lithium salt, β-Nicotinamide adenine dinucleotide 2′-phosphate reduced tetrasodium salt hydrate (NADPH), β-Nicotinamide adenine dinucleotide phosphate sodium salt hydrate (NADP+), D-Glucose 6-phosphate sodium salt, ethylene glycol monomethyl ether, fluorescein, heparin sodium salt, iodoacetamide, L-malic acid sodium salt, malonyl Coa lithium salt, phenylmethylsulphonyl fluoride (PMSF) and triethanolamine hydrochloride were all purchased from Sigma-Aldrich (St. Louis, MO, USA). Fluorescein dibutyrate was obtained from ICN Biomed (London, UK).

Primary antibodies anti-FAS, -DGAT2, -PLIN1 and -Vinculin were obtained from Abcam (Cambridge, UK). Primary antibodies anti-FATP1 (SLC27A1), -ABHD5 (CGI-58) and -pHSL (Ser 660) were purchased from Thermo Fisher Scientific (Waltham, MA, USA). Primary antibodies anti-total HSL, -CD36, -ATGL, -pACC and -total ACC were obtained from Cell Signalling Technology (Danvers, MA, USA). Primary antibodies anti-AQP9, -AQP7 and -GLUT4 were purchased from Santa Cruz Biotechnology (Dallas, TX, USA).

### 2.2. Opuntia stricta var. dillenii Extract

Fruits of *Opuntia stricta* var. *dillenii* were harvested on Tenerife Island in the Canary Archipelago, Spain (28°32′03″ N, 16°23′50″ W above sea level) [18]. The prickly pear peel was subjected to a freezing and drying process, followed by repeated extractions with methanol:water (1:1, *v*:*v*) mixture and methanol. Samples were extracted one last time with methanol and supernatants were evaporated in a rotary evaporator. After that, aqueous extracts were made with pure water, resulting in an aqueous extract rich in betalains and phenolic compounds [19]. The composition of the extract, analysed by HPLC, is shown on Table 1. After freezing–drying the extract, a stock solution of 200 mg/mL was prepared by dissolving it in water, then aliquoted and stored at −20 °C until administration to the rats.

### 2.3. Animals, Diets, and Experimental Design

For this study, five-week-old male Wistar rats (n = 40; 120–130 g) were purchased from Envigo (Barcelona, Spain). Rats were housed in polycarbonate metabolic cages in a temperature-controlled room (22 ± 2 °C) with a 12 h light/dark cycle (lights on from 23:00 to 11:00). After a six-day adaptation period, animals were randomly assigned to four experimental groups (n = 10 per group): the control group (C) received a commercial standard diet (AIN-93G, OpenSource Diets, New Brunswick, NJ, USA, D10012G); the HFHF group was fed a high-fat high-fructose diet containing 40% fat and 10% fructose (OpenSource Diets, New Brunswick, NJ, USA, D09100310); animals in the OD groups were fed the same high-fat high-fructose diet supplemented with an *Opuntia stricta* var. *dillenii* peel extract, at doses of 25 mg extract/kg body weight/day in ODL group (low dose), and of 100 mg extract/kg body weight/day in ODH group (high dose). The choice of doses used in this in vivo study was based on the available literature; in the reported studies the doses of *Opuntia* extracts were in the range of 25 to 300 mg/kg body weight/day, being the dose of 100 mg/kg body weight/day the most commonly used [13]. The experimental extract was administered orally to the rats directly via pipette once daily for 8 weeks, in an oral solution containing 2.5% sucrose. Non-treated animals (C and HFHF groups) also received this vehicle. Rats were fed ad libitum and had free access to water. Food intake and body weight were recorded daily. Table 2 summarises the nutrient composition of the experimental diets used.

**Figure 2:**
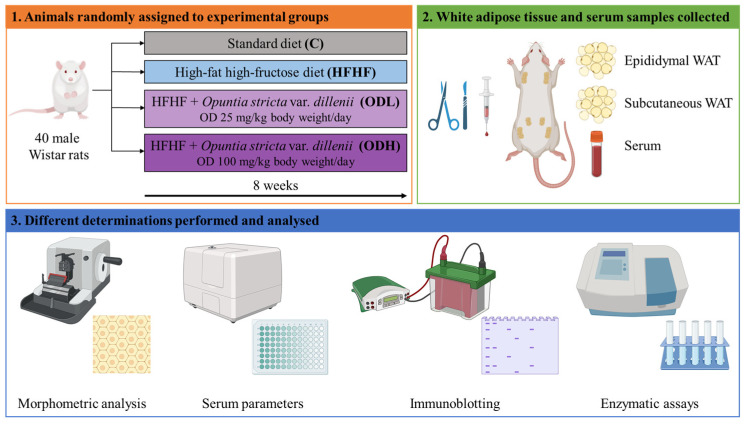
Schematic representation of the experimental design (Created using BioRender, https://BioRender.com).

At the end of the eight-week experimental period, animals were euthanised under anaesthesia (chloral hydrate) and sacrificed by cardiac exsanguination following a 12 h fast. White adipose tissue from various anatomical locations (epididymal, perirenal, mesenteric, and subcutaneous) was dissected, weighed and immediately frozen in liquid nitrogen (Figure 2). All samples were stored at −80 °C until analysis. All procedures were conducted in accordance with the guidelines of the Ethical Committee of the University of the Basque Country for the care and use of laboratory animals (M20_2022_283).

### 2.4. Serum Parameters

After centrifugation of blood samples for ten minutes at 1000× *g* and 4 °C, serum was aliquoted for biochemical analysis. Serum high-density lipoprotein- (HDL-), non-HDL- and total-cholesterol levels were determined using commercial kits from BioSystems (Barcelona, Spain). Triacylglycerides and non-esterified fatty acid (NEFA) concentrations were measured using kits from Spinreact (Barcelona, Spain) and Fujifilm (Lexington, MA, USA), respectively. Adiponectin and leptin determinations were quantified using ELISA kits (Merck KGaA, Darmstadt, Germany).

### 2.5. Adipocyte Morphometric Analysis

Morphometric analyses were conducted on epididymal and subcutaneous white adipose tissue samples. Tissues were fixed in 4% paraformaldehyde and embedded in paraffin, following a previously described protocol [20]. Sections of 5 μm thickness were obtained using a microtome, mounted on slides, and stained with haematoxylin/eosin. Tissue sections were examined with a light microscope (Zeiss Axioskop 2 microscope) equipped with an AxioCam ICc3 digital camera (Carl Zeiss, S.A., Barcelona, Spain). White adipocytes area of 8 animals per group (mean of around 180 cells/group) was measured using Zen 3.4 (blue edition) software from Zeiss. Distributions of adipocyte size were obtained from individual data of cell sizes.

### 2.6. Immunoblotting for Protein Expression Measurement

For the analysis of abhydrolase domain containing 5-lysophosphatidic acid acyltransferase (ABHD5, also known as CGI-58), acetyl-CoA carboxylase (ACC), adipose triglyceride lipase (ATGL), aquaporin 7 (AQP7), aquaporin 9 (AQP9), cluster of differentiation 36 (CD36), diacylglycerol O-acyltransferase 2 (DGAT2), fatty acid synthase (FAS), fatty acid transport protein 1 (FATP1), glucose transporter 4 (GLUT4), hormone-sensitive lipase (HSL), perilipin 1 (PLIN1), phosphorylated acetyl-CoA carboxylase (pACC), phosphorylated hormone-sensitive lipase (pHSL) and vinculin, total protein extraction was conducted. Specifically, 500 mg of epididymal and 400 mg of subcutaneous tissue were homogenised in 700 µL of cellular PBS buffer (pH 7.4) containing protease inhibitors. Homogenization was performed using 5 s bursts at 60% amplitude with a Branson SFX550 Sonifier (Saint Louis, MO, USA) equipped with a microtip, followed by centrifuging for five minutes (800× *g*, 4 °C). Total protein content was quantified spectrophotometrically at 595 nm using the Bradford method [21], with bovine serum albumin (BSA) as the standard.

Immunoblotting assays were conducted using 30 μg of total protein from adipose tissue homogenates, denatured for five minutes at 95 °C in Laemmli buffer [22] and separated by SDS-PAGE using 4–15% precast gels. The samples were transferred onto PVDF membranes by electroblotting at a constant intensity (1 mA/cm^2^). After transfer, the membranes were blocked with 4% BSA in PBS-Tween buffer for two hours at room temperature. The membranes were then blotted overnight at 4 °C with the appropriate primary antibodies: CGI-58 (1:1000), ACC (1:1000), ATGL (1:1000), AQP7 (1:1000), AQP9 (1:1000), CD36 (1:1000), DGAT2 (1:1000), FAS (1:1000), FATP1 (1:1000), GLUT4 (1:1000), HSL (1:500), PLIN1 (1:1000), p-ACC (1:1000), p-HSL (1:500) and vinculin (1:1000). Membranes were subsequently incubated with a horseradish peroxidase-conjugated secondary antibody. Proteins were detected using the Supersignal West Femto Maximum Sensitivity substrate (ThermoScientific, Waltham, MA, USA) and the blots were imaged with the ChemiDoc^TM^ MP Imaging System scanner (Bio-Rad; Hercules, CA, USA). Vinculin was used as the housekeeping protein.

### 2.7. Enzyme Activity in Adipose Tissue

For the analysis of lipogenic enzymes, samples of epididymal (500 mg) and subcutaneous (400 mg) adipose tissue were homogenised in 700 µL and 1 mL of buffer, respectively, containing 10 mM Tris HCl, 250 mM sucrose and 1 mM EDTA (pH 7.4). The homogenates were then centrifuged at 700× *g* for ten minutes at 4 °C. Subsequently, the supernatants were collected and further centrifuged at 12,000× *g* for 15 min at 4 °C. The resulting supernatant fraction was used for the quantitation of enzyme activities. Fatty acid synthase (FAS), glucose-6-phosphate dehydrogenase (G6PD), and malic enzyme (ME) activities were measured as previously described [23]. Enzyme activity was expressed as nanomoles of NADPH consumed (for FAS) or produced (for G6PD and ME) per minute per milligram of protein.

For the lipoprotein lipase activity (LPL), heparin-releasable LPL was measured using fluorometric method. In total, 200 mg of both epididymal and subcutaneous adipose tissue were incubated in 1 mL of Krebs–Ringer–Phosphate buffer containing 0.15 M NaCl, KCl and MgSO_4_, 0.10 M CaCl_2_, and sodium heparin at 37 °C for 45 min. All samples were then re-incubated in phosphate buffer containing fluorescein dibutyrate and ethylene glycol monomethyl ether for five minutes, after which the reaction was stopped by placing the samples on ice. Fluorescence was measured at 490 nm excitation and 530 nm emission. Enzymatic activity was expressed as nanomoles of produced fluorescence per minute per gram of tissue. Protein quantity was measured in all cases using the Bradford method [21].

### 2.8. Statistical Analysis

Results are presented as the mean ± standard error of the means (SEM). Statistical analysis was performed using SPSS 25.0 (SPSS, Chicago, IL, USA). The normality of the data distribution was assessed using the Shapiro–Wilk’s test. Data were analysed by one-way ANOVA followed by the Newman–Keuls post hoc test. Statistical significance was set at the *p* < 0.05 level.

## 3. Results

### 3.1. Food and Energy Intake, Total Body Weight, Adipose Pad Weights, and White Adipose Index

At the end of the experimental period, no changes in food intake were found among groups (Table 3). Nevertheless, since the HFHF diet provided a higher amount of energy compared to the control, rats fed the obesogenic diet (HFHF, ODL, and ODH) showed significantly higher energy intake (*p* < 0.05). Total body weight increased markedly in the HFHF group compared to rats fed a standard diet (*p* < 0.05). Moreover, this feeding pattern provoked a significant increase in the weight of all adipose tissues measured (epididymal, perirenal, mesenteric and subcutaneous), as well as in total white adipose tissue. The obesogenic diet also led to an increase in the white adipose tissue index, defined as the percentage of total white adipose tissue relative to body weight. *Opuntia* supplementation at the low dose (ODL) partially prevented the rise in total white adipose tissue weight and the combined weight of visceral tissues, and it was also associated with a lower white adipose index. At a high dose (ODH), *Opuntia* supplementation significantly prevented the increase in the weight of epididymal and mesenteric adipose pads, as well as total and visceral white adipose tissue. Although the reduction did not reach statistical significance, subcutaneous pad weight was 13% lower in both ODL (*p* = 0.06) and ODH (*p* = 0.07) groups compared to the HFHF cohort.

### 3.2. Serum Biochemical Parameters

Concerning serum lipids, no change in triglyceride concentration was observed between the HFHF and C groups (Table 4). In terms of *Opuntia* treatments, triglyceride levels were significantly reduced in the ODL group compared to the control group (*p* < 0.05), while the ODH group showed a significant reduction compared to both the HFHF (*p* < 0.01) and C groups (*p* < 0.001).

Concerning cholesterol, the HFHF group exhibited significantly higher level of total cholesterol (TC) serum concentration, compared to the controls (*p* < 0.001). This increase was partially prevented by the ODL extract (*p* < 0.05), while no significant differences were observed between the ODH and HFHF groups. The obesogenic diet did not affect HDL-C serum concentration; however, the high dose of *Opuntia* extract significantly increased HDL-C levels, compared to the HFHF group (*p* < 0.01). Non-HDL-C concentration was higher in the HFHF group in comparison to the controls (*p* < 0.001). The low dose of the extract, but not the high dose, partially prevented this increase (*p* < 0.05). Lastly, NEFA serum concentrations remained unchanged across all groups, and no differences in adiponectin and leptin concentrations were observed.

### 3.3. Histological Analysis

**Figure 3:**
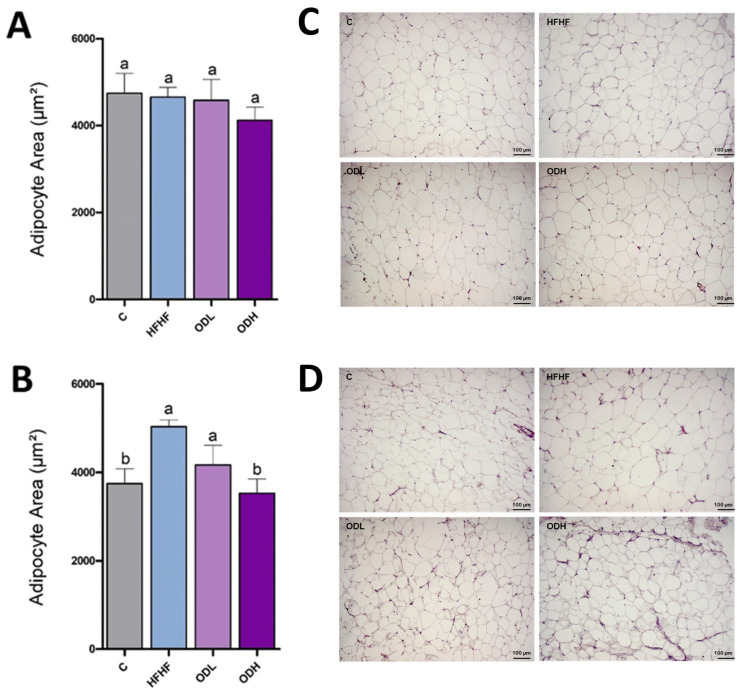
Adipocyte morphometry analysis. Mean adipocyte area in epididymal (**A**) and subcutaneous (**B**) white adipose tissue and representative sections illustrating adipocyte size in epididymal (**C**) and subcutaneous (**D**) depots from rats fed a standard diet (C group), a high-fat high-fructose diet (HFHF), a high-fat high-fructose diet supplemented with *Opuntia stricta* var. *dillenii* peel extract at a dose of 25 mg/kg body weight/day (ODL group) or a high-fat high-fructose diet supplemented with *Opuntia stricta* var. *dillenii* peel extract at 100 mg/kg body weight/day (ODH group) for eight weeks. The area of individual adipocytes (μm^2^) was measured using a quantitative morphometry at 10× magnification using Zen 3.4 (blue edition) software from Zeiss. A total of 8 animals per group, and a mean value of 180 cells per group, were analyzed. The values are presented as mean ± SEM. Bars not sharing a common letter are significantly different (*p* < 0.05).

To assess potential morphological changes in white adipose tissue induced by the *Opuntia* cactus extract, histological analysis was conducted in epididymal and subcutaneous fat depots (Figure 3). In the epididymal tissue, adipocyte size was similar in the C, HFHF and ODL groups. However, in the group treated with the high dose of the extract (ODH group), a tendency towards reduced cell size values vs. the HFHF was observed (*p* = 0.08). In the subcutaneous depot, a significant increase in adipocyte size was triggered by the obesogenic diet, in comparison with rats fed a standard diet. Regarding the treated groups, both doses of the extract reduced adipocyte area (by 17% and 30% for the low and the high doses, respectively), although statistical significance was reached only in the ODH group.

**Figure 4:**
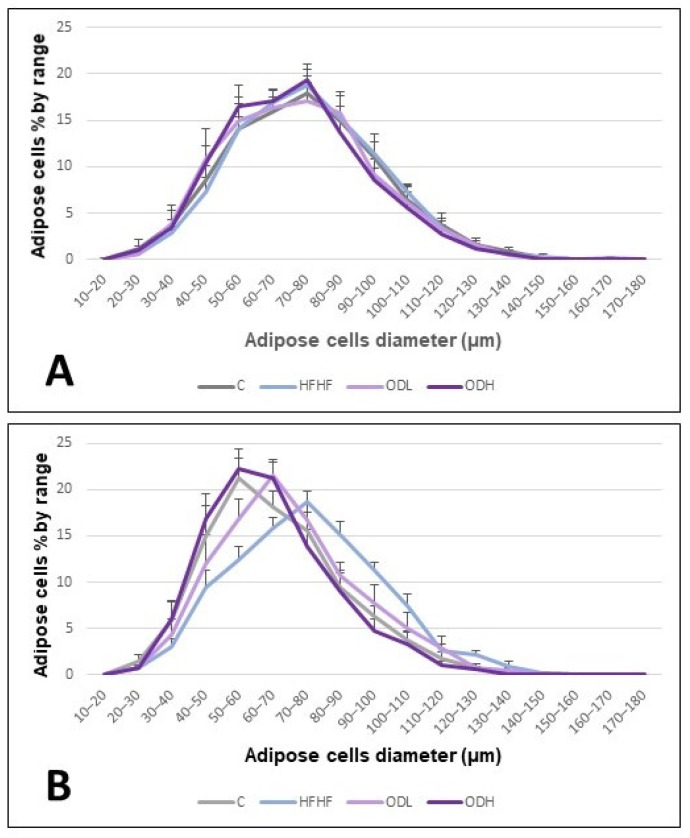
Adipocyte size distribution analysis in epididymal (**A**) and subcutaneous (**B**) white adipose tissue from rats fed a standard diet (C group), a high-fat high-fructose diet (HFHF), high-fat high-fructose diet supplemented with *Opuntia stricta* var. *dillenii* peel extract at a dose of 25 mg/kg body weight/day (ODL group) or a high-fat high-fructose diet supplemented with *Opuntia stricta* var. *dillenii* peel extract at 100 mg/kg body weight/day (ODH group) for eight weeks. Distributions of adipose cells sizes across the fat depots were obtained from individual cell area measurements including all animals (n = 10) in each group.

Differences in adipose cell size distribution were also analysed (Figure 4). In the subcutaneous depot, the obesogenic diet caused a shift toward a higher proportion of larger adipocytes compared to control rats. This effect appeared to be normalised by high-dose extract supplementation, and, to a lesser extent, by the low-dose extract. In contrast, no noticeable differences in adipocyte size distribution were detected among groups in the epididymal adipose tissue.

### 3.4. Protein Expression and Enzymatic Activities in Epididymal Tissue

**Figure 5:**
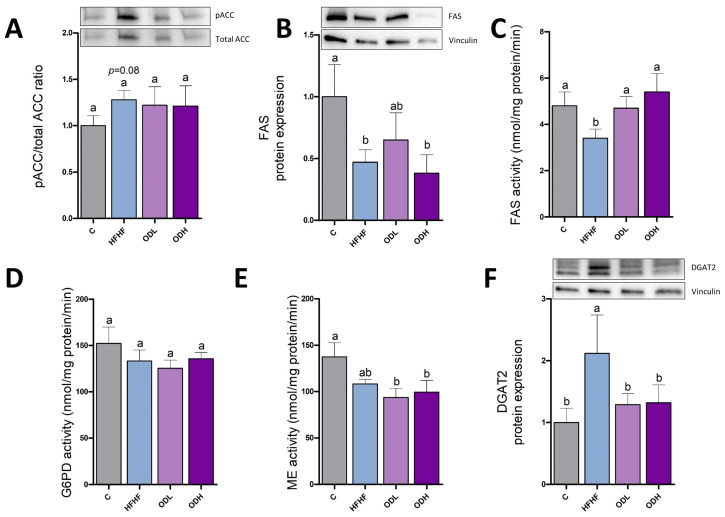
Relative protein levels of pACC to ACC (pACC/ACC ratio) (**A**), protein expression of FAS (**B**), enzymatic activities of FAS (**C**), G6PD (**D**) and ME (**E**), and protein expression of DGAT2 (**F**) in epididymal white adipose tissue from rats (n = 10 animals per group) fed standard diet (C group), a high-fat high-fructose diet (HFHF), high-fat high-fructose diet supplemented with *Opuntia stricta* var. *dillenii* peel extract at a dose of 25 mg/kg body weight/day (ODL group) or a high-fat high-fructose diet supplemented with *Opuntia stricta* var. *dillenii* peel extract at 100 mg/kg body weight/day (ODH group) for eight weeks. The values are presented as mean ± SEM. Bars not sharing a common letter are significantly different (*p* < 0.05). ACC: acetyl-coA carboxylase; DGAT2: diacylglycerol O-acyltransferase 2; FAS: fatty acid synthase; G6PD: glucose-6-phosphate dehydrogenase; ME: malic enzyme; pACC: phosphorylated acetyl-coA carboxylase. The total protein was used for protein normalization in phosphorylated/total ACC ratio in western blotting.

In order to analyse the effects of *Opuntia* dietary supplementation on the de novo lipogenesis pathway, pACC/ACC ratio was measured as an index of the enzyme activity, given that this enzyme is inhibited by phosphorylation (Figure 5A). Moreover, FAS protein expression and activity were assessed (Figure 5B,C). These two enzymes play a key role in fatty acids synthesis, a crucial process for energy storage in the form of triglycerides. A trend (*p* = 0.08) to a greater pACC/ACC ratio (28% of increase) was evident in the epididymal adipose tissue of the rats fed a high-fat high-fructose diet, compared to those fed a standard diet. In addition, a significant decrease in both protein expression and activity of FAS was observed in the HFHF group in comparison with the control group. Additionally, the enzymatic activities of G6PD and ME—two key enzymes involved in the synthesis of the NADPH required for de novo lipogenesis—, were also assessed (Figure 5D,E). No changes in these enzymes were induced by high-fat high-fructose feeding. Concerning the *Opuntia stricta* var. *dillenii* treatments, no changes were observed in these enzymes, with the exception of FAS. In this case, both doses of the *Opuntia* extract totally prevented the decrease in the activity induced by high-fat high-fructose feeding. To analyse the final step of triglyceride assembly, the expression of the DGAT2 protein was further assessed (Figure 5F). A sharp increase in DGAT2 levels was observed in animals fed the high-fat high-fructose diet compared to controls (*p* < 0.05). This boost was completely prevented by both doses of *Opuntia* extract.

**Figure 6:**
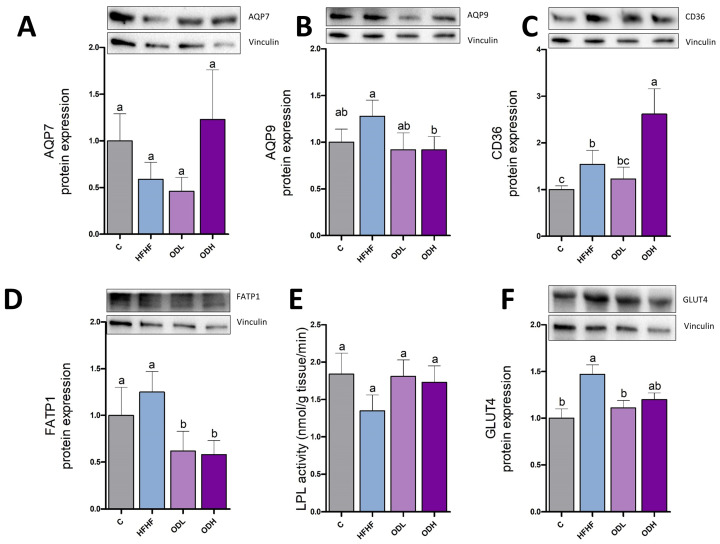
Protein expression of AQP7 (**A**), AQP9 (**B**), CD36 (**C**), FATP1 (**D**), enzymatic activity of LPL (**E**) and protein expression of GLUT4 (**F**) in epididymal white adipose tissue from rats (n = 10 animals per group) fed standard diet (C group), a high-fat high-fructose diet (HFHF), high-fat high-fructose diet supplemented with *Opuntia stricta* var. *dillenii* peel extract at a dose of 25 mg/kg body weight/day (ODL group) or a high-fat high-fructose diet supplemented with *Opuntia stricta* var. *dillenii* peel extract at 100 mg/kg body weight/day (ODH group) for eight weeks. The values are presented as mean ± SEM. Bars not sharing a common letter are significantly different (*p* < 0.05). AQP7: aquaporin 7; AQP9: aquaporin 9; CD36: cluster of differentiation 36; FATP1: fatty acid transport protein 1; GLUT4: glucose transporter 4; LPL: lipoprotein lipase.

The effects of the treatment on glycerol and fatty acids transport were also studied, by measuring the expression of aquaglyceroporins (AQP7 and AQP9), CD36, and FATP1 (Figure 6A–D). AQP7 protein expression, involved in glycerol efflux, decreased by 41% in animals fed the high-fat high-fructose diet, compared to controls, although this decline did not reach statistical significance due to high variability in the groups (*p* = 0.1). AQP9, which facilitates glycerol entry into adipocytes, remained unchanged. Finally, CD36 expression was significantly raised in response to the obesogenic diet. In the case of *Opuntia* extract, the low dose only modified FATP1 transporter, inducing a decrease even if this protein remained unchanged in the HFHF group. As far as the high dose is concerned, it induced a significant increase in CD36 expression and significant decreases in AQP9 and FATP1.

To measure the uptake of fatty acids from triglycerides circulating as lipoproteins, LPL activity was assessed (Figure 6E). No significant difference was observed among the experimental groups in this parameter. To assess glucose uptake into the adipocytes, GLUT4 protein expression was measured (Figure 6F). The obesogenic diet significantly increased GLUT4 expression compared with control rats. This increase was only prevented by the low dose of the extract.

**Figure 7:**
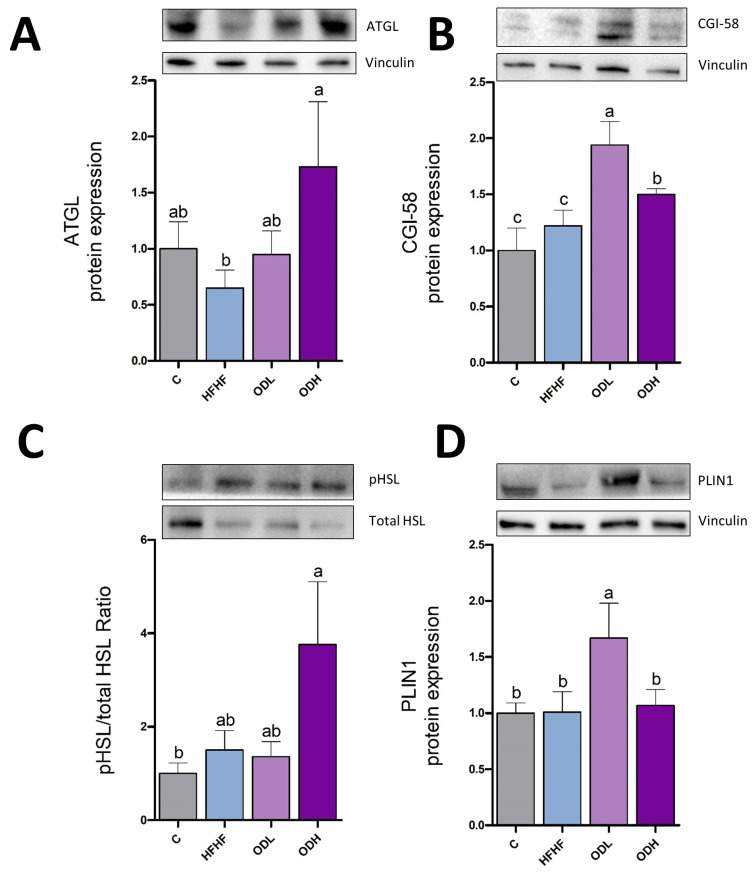
Protein expression of ATGL (**A**), CGI-58 (**B**), pHSL/HSL ratio (**C**) and PLIN1 (**D**) in epididymal white adipose tissue from rats (n = 10 animals per group) fed a standard diet (C group), a high-fat high-fructose diet (HFHF), a high-fat high-fructose diet supplemented with *Opuntia stricta* var. *dillenii* peel extract at a dose of 25 mg/kg body weight/day (ODL group) or a high-fat high-fructose diet supplemented with *Opuntia stricta* var. *dillenii* peel extract at 100 mg/kg body weight/day (ODH group) for eight weeks. The values are presented as mean ± SEM. Bars not sharing a common letter are significantly different (*p* < 0.05). CGI-58: 5-lysophosphatidic acid acyltransferase; ATGL: adipose triglyceride lipase; HSL: hormone-sensitive lipase; PLIN1: perilipin 1; pHSL: phosphorylated hormone-sensitive lipase. The total protein was used for protein normalization in phosphorylated/total HSL ratio in western blotting.

Since lipolytic enzymes are key regulators of stored lipid breakdown, the expression of ATGL and the activation of HSL (pHSL/HSL ratio), as well as protein expression of CGI-58 and PLIN1 were measured (Figure 7). ATGL initiates lipolysis by hydrolysing triglycerides into diacylglycerol and free fatty acids, while CGI-58 enhances ATGL activity by facilitating its interaction with lipid droplets and promoting efficient lipolysis. No changes were induced by the obesogenic diet in these parameters. HSL, the lipase that acts after ATGL by hydrolyzing diglycerides was not modified by high-fat high-fructose feeding. Lastly, PLIN1, which regulates lipid droplet dynamics by restricting lipase access to stored triglycerides and acts as a protective layer around lipid droplets, also remained unchanged. Regarding *Opuntia* extract, whereas the low dose significantly increased protein expression of CGI-58 and PLIN-1, without changes in both lipases, the high dose significantly increased protein expression of ATGL and CGI-58 and produced a trend towards higher HSL activity levels (+150%, *p* = 0.07), without changes in PLIN-1.

### 3.5. Protein Expression and Enzymatic Activities in Subcutaneous Tissue

**Figure 8:**
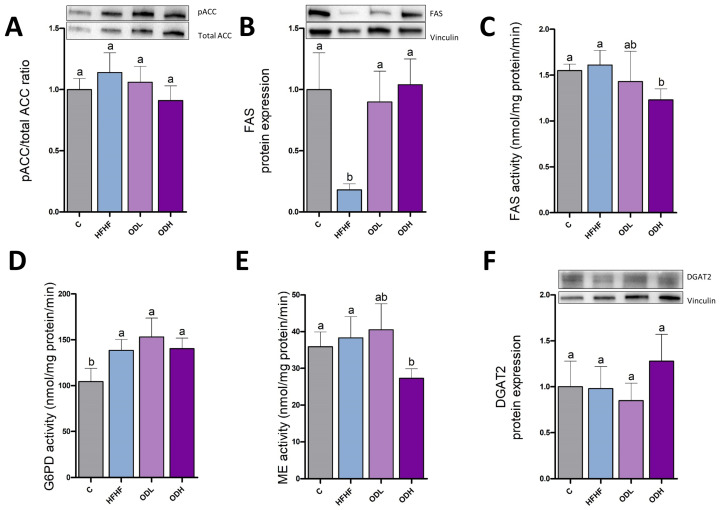
Relative protein levels of pACC to ACC (pACC/ACC ratio) (**A**), protein expression of FAS (**B**), enzymatic activities of FAS (**C**), G6PD (**D**) and ME (**E**), and protein expression of DGAT2 (**F**) in subcutaneous white adipose tissue from rats (n = 10 animals per group) fed a standard diet (C group), a high-fat high-fructose diet (HFHF), a high-fat high-fructose diet supplemented with *Opuntia stricta* var. *dillenii* peel extract at a dose of 25 mg/kg body weight/day (ODL group) or a high-fat high-fructose diet supplemented with *Opuntia stricta* var. *dillenii* peel extract at 100 mg/kg body weight/day (ODH group) for eight weeks. The values are presented as mean ± SEM. Bars not sharing a common letter are significantly different (*p* < 0.05). ACC: acetyl-coA carboxylase; DGAT2: diacylglycerol O-acyltransferase 2; FAS: fatty acid synthase; G6PD: glucose-6-phosphate dehydrogenase; ME: malic enzyme; pACC: phosphorylated acetyl-coA carboxylase. The total protein was used for protein normalization in phosphorylated/total ACC ratio in western blotting.

The same protein expressions and activities were measured in subcutaneous adipose tissue. For lipogenic proteins, no changes in pACC/ACC ratio were observed among groups. In contrast, FAS expression showed a very marked reduction in the HFHF group compared to controls. This decrease was significantly prevented by both extracts (Figure 8A,B). This modification was not reflected in FAS activity, which was significantly reduced in ODH group compared to HFHF group. The obesogenic diet increased G6PD activity but did not modify ME. Nonetheless, significant decreases in FAS and ME activities were observed in rats supplemented with the high-dose extract. In the case of G6PD activity, a significant increase was observed in all high-fat high-fructose diet-fed groups compared to controls, with no effect attributable to extract administration (Figure 8C–E). No differences in DGAT2 expression were detected among the experimental groups (Figure 8F).

**Figure 9:**
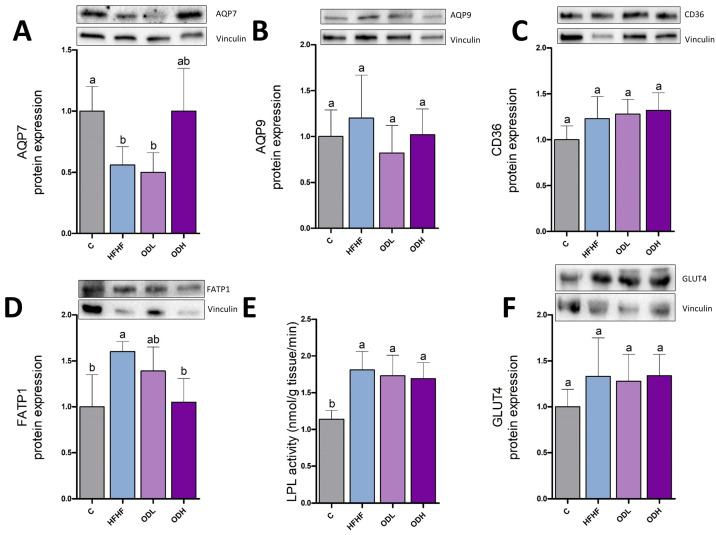
Protein expression of AQP7 (**A**), AQP9 (**B**), CD36 (**C**), FATP1 (**D**), enzymatic activity of LPL (**E**), and protein expression of GLUT4 (**F**) in subcutaneous white adipose tissue from rats (n = 10 animals per group) fed a standard diet (C group), a high-fat high-fructose diet (HFHF), a high-fat high-fructose diet supplemented with *Opuntia stricta* var. *dillenii* peel extract at a dose of 25 mg/kg body weight/day (ODL group) or a high-fat high-fructose diet supplemented with *Opuntia stricta* var. *dillenii* peel extract at 100 mg/kg body weight/day (ODH group) for eight weeks. The values are presented as mean ± SEM. Bars not sharing a common letter are significantly different (*p* < 0.05). AQP7: aquaporin 7; AQP9: aquaporin 9; CD36: cluster of differentiation 36; FATP1: fatty acid transport protein 1; GLUT4: glucose transporter 4; LPL: lipoprotein lipase.

In the subcutaneous fat depot, the high-fat high-fructose diet significantly reduced AQP7 expression. The low-dose extract produced no changes in comparison with the HFHF group, while the high-dose extract totally prevented the AQP7 decrease induced by the high-fat high-fructose diet, because this group reached the same value than the control group. Nevertheless, the statistical analysis only revealed a tendency (*p* = 0.1) due to the high value of SEM. No differences in AQP9 and CD36 protein expression were observed among all experimental groups. A higher FATP1 expression level was seen in the HFHF group compared to controls (*p* < 0.05). The low-dose extract prompted no changes in FATP1 expression in comparison with the HFHF group, whereas the high-dose extract significantly prevented the diet-induced increase (Figure 9A–D).

Regarding LPL activity, a significant rise was triggered by the obesogenic diet; the extract did not prevent this increase (Figure 9E). No changes in GLUT4 expression were observed among the experimental groups. (Figure 9F).

**Figure 10:**
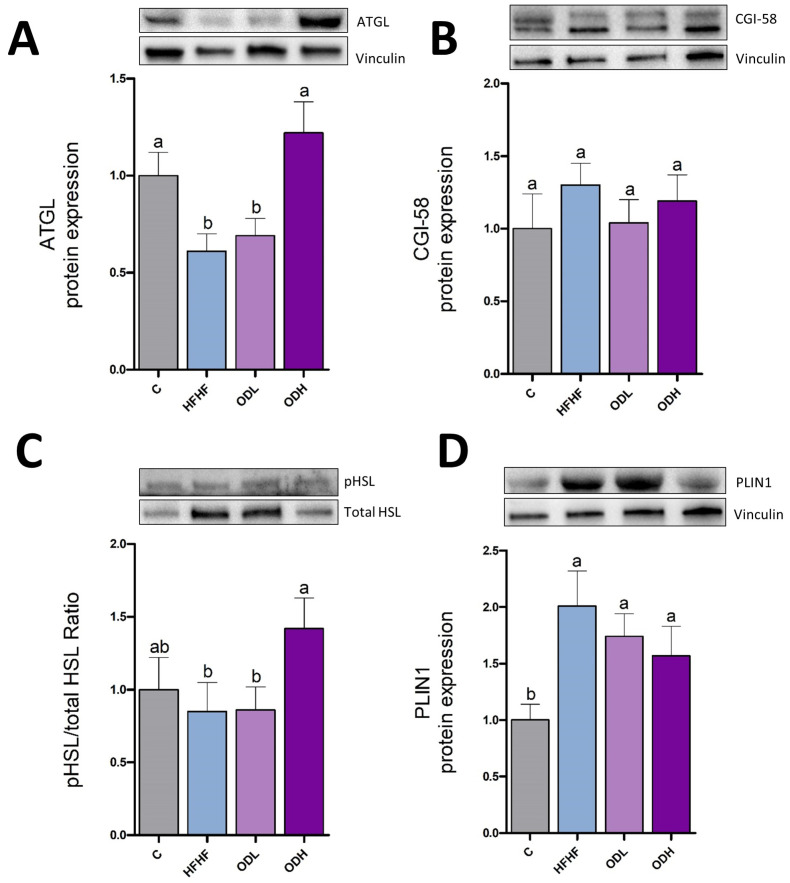
Protein expression of ATGL (**A**), CGI-58 (**B**), pHSL/HSL ratio (**C**) and PLIN1 (**D**) in subcutaneous white adipose tissue from rats (n = 10 animals per group) fed a standard diet (C group), a high-fat high-fructose diet (HFHF), a high-fat high-fructose diet supplemented with *Opuntia stricta* var. *dillenii* peel extract at a dose of 25 mg/kg body weight/day (ODL group) or a high-fat high-fructose diet supplemented with *Opuntia stricta* var. *dillenii* peel extract at 100 mg/kg body weight/day (ODH group) for eight weeks. The values are presented as mean ± SEM. Bars not sharing a common letter are significantly different (*p* < 0.05). CGI-58: 5-lysophosphatidic acid acyltransferase; ATGL: adipose triglyceride lipase; HSL: hormone-sensitive lipase; PLIN1: perilipin 1; pHSL: phosphorylated hormone-sensitive lipase. The total protein was used for protein normalization in phosphorylated/total HSL ratio in western blotting.

Regarding the lipolytic enzymes, a sharp decrease in ATGL protein expression (-39%) was observed in the HFHF group compared to controls (*p* < 0.01). The administration of the *Opuntia* extract at the highest dose prevented this reduction, restoring ATGL expression to control levels, while no change was seen in the ODL group. No differences in CGI-58 expression levels were found among the experimental groups. As for HSL expression, no changes in the phosphorylated-HSL/HSL ratio induced by the high-fat high-fructose diet were observed. The high-dose *Opuntia* extract significantly increased HSL expression by 66%. Lastly, a marked PLIN1 expression boost (+100%) was observed in rats fed the high-fat high-fructose diet (*p* < 0.01), while no changes were observed in the ODL and ODH groups compared to the HFHF group (Figure 10).

## 4. Discussion

Obesity is a major contributor to several chronic metabolic diseases, and the rise in its prevalence is posing a significant global health challenge. In this context, excessive consumption of unbalanced diets rich in sugars and saturated fats is associated with increased energy intake and body weight, thereby elevating the risk of developing obesity [24]. This has prompted growing interest in the use of plant-derived compounds as a strategy to manage obesity and its related diseases [25,26]. Research into extracts obtained from plants or their by-products is expanding, as these substances exhibit anti-obesity properties and may represent a more cost-effective alternative to synthetic compounds [27,28]. In this context, the present study aims to investigate whether an *Opuntia stricta* var. *dillenii* peel extract, rich in betalains and phenolic compounds, is able to prevent obesity induced by a high-fat high-fructose diet in rats. For this purpose, the extract was administered concurrently with the high-fat high-fructose diet. The peel extract was selected for administration due to its ability to significantly reduce triglyceride content in 3T3-L1 mature adipocytes, as previously described by our group [29]. Additionally, the peel extract of *Opuntia stricta* var. *dillenii* was the most effective in preventing steatosis in an in vitro model of hepatic fatty liver [30]. Moreover, it presents an interesting option within the context of sustainability and the circular economy, as it is derived from food industry waste.

In the present study, feeding rats a diet rich in saturated fats and fructose led to an increase in body weight and greater fat pad mass across all dissected adipose tissues, confirming the effectiveness of this dietary pattern in establishing a murine model of obesity. The treatment of animals fed the high-fat high-fructose diet with two different doses (25 mg/kg body weight/day; ODL group, or 100 mg/kg body weight/day; ODH group) of the extract of *Opuntia stricta* var. *dillenii* peel fruit resulted in partial prevention of the obesity development. Indeed, significantly decreased values of total adipose tissue weights and visceral adipose tissue weights were observed in both ODL and ODH groups. The subcutaneous depot showed in these groups an intermediate value between the control and the HFHF rats, but the decrease (−13.1%) did not reach statistical significance. Despite the reductions in visceral adipose tissues, final body weight remained unchanged in the groups treated with *Opuntia* extracts. This can be due to the fact that subcutaneous adipose tissue was not significantly reduced in the present study, being another large portion of total body fat. On the other hand, a reduction that reaches statistical significance in adipose tissue, due to its relatively small size, loses such significance when evaluating the total body weight of the animal, which at the time of sacrifice was in the 450–500 g range. Finally, increases in muscle mass and/or body water cannot be discarded.

For the histological analysis, the epididymal and the subcutaneous depots were selected. Notably, the effects of the *Opuntia* extract on adipose tissue morphology differed between the two depots. In the epididymal tissue, although high-fat high-fructose feeding led to a significant increase in tissue mass, no changes in adipocyte size were observed, suggesting a possible increase in adipocyte number (hyperplasia) or in other components such as stromal or inflammatory cells. Although the epididymal depot generally tends to expand via hypertrophy, this aligns with previous studies in rats, which have shown that the epididymal depot can undergo hyperplasia under specific conditions, particularly during intermediate phases of high-fat diet feeding [31]. With the intervention, adipocyte size tended to be smaller in the ODH group, a shift that aligns with the overall decrease in epididymal adipose tissue weight. However, this reduction may be better attributed to the prevention of the increase in the number of adipocytes rather than the shrinkage of individual cells. In contrast, in the subcutaneous depot, the increase in tissue mass associated with intake of the high-fat high-fructose diet was accompanied by an increase in adipocyte size. In this depot, the high dose of the extract prevented adipocyte hypertrophy induced by high-fat high-fructose feeding. Moreover, the high dose, and to a lesser extent the low dose, increased the number of smaller adipocytes, resulting in a size distribution pattern more comparable to that of the control group. However, despite these changes observed at the cellular level, no significant reduction in overall subcutaneous adipose tissue size was observed. It is possible that the treatment duration was insufficient to produce measurable changes in tissue mass, or that inter-individual variability limited the ability to detect statistically significant differences. The increased number of smaller adipocytes is highly relevant, since adipocyte hypertrophy can be accompanied by fibrosis, hypoxia and inflammation of the tissue [32].

The outcomes of the present investigation cannot be directly compared with existing literature, as no previous studies have examined the effects of *Opuntia stricta* var. *dillenii* cactus peel extract on obesity. Available research has focused primarily on other *Opuntia* species, particularly *Opuntia ficus-indica.* Moreover, most studies have not utilised fruit peel extracts but rather other plant parts, mainly cladodes. Nevertheless, comparisons among different *Opuntia* species or among different plant parts should be made with caution because important differences can be found in the bioactive compound profile. Thus, the peel of *Opuntia stricta* var. *dillenii* presents a high content of betacyanins (betanin, isobetanin, phyllocactin and neobetanin), as well as piscidic acid. In contrast, *Opuntia ficus indica* is rich in piscidic acid and indicaxanthin, being the latter not detected in *Opuntia stricta* var. *dillenii*. Furthermore, antinutritional factors, which may affect the bioavailability of nutrients or interfere with the bioactive compounds, are commonly found in food by-products such as fruit peel. Certain phytochemical analyses have detected antinutritional factors such as oxalates, tannins, phytates, and saponins in the pulp and seeds of cacti from the genus *Opuntia* [33,34]; unfortunately, there is no available literature on potential antinutrients in the peel of *Opuntia stricta* var. *dillenii*. Concerning the main bioactive compounds present in the extract, betanin, the predominant compound in this *Opuntia* variety, has been reported to exert potential anti-obesity effects [35,36]. In addition, isorhamnetin glycosides have also demonstrated anti-obesity activity by reducing adipocyte size in visceral and subcutaneous depots in a dietary model of obesity [37].

Obesity is commonly associated with alterations in serum lipids. Indeed, in the present experimental model, an increase in total cholesterol and non-HDL cholesterol levels was observed in HFHF group. Regarding the low dose of *Opuntia stricta* var. *dillenii* peel extract, it was able to partially prevent the diet-induced increase in total cholesterol and non-HDL cholesterol levels. Moreover, the high dose was able to increase HDL-cholesterol level and to reduce triglyceride level. The reduction in serum triglycerides is in line with the results provided by Bouhrim et al., (2018) administering *Opuntia dillenii* seed oil for two weeks to Wistar rats; these authors showed a reduction in the increase caused by CCl_4_ in serum triglyceride content [38]. These positive changes in serum profile were not mirrored by changes in leptin or adiponectin plasma concentrations. It is known that changes in adipokine levels tend to require longer intervention periods to become evident, particularly as compared to serum lipid parameters, which typically respond more rapidly to dietary treatments [39].

The remaining results cannot be compared with literature because no data coming from other studies are available. It is well known that visceral, but not subcutaneous adipose tissue, is related to metabolic alterations such as dyslipidemia [40]. Thus, the hypolipidemic effect of the *Opuntia* extract is in good accordance with the reduced size of visceral fat depots.

To elucidate the mechanisms underlying the obesogenic effect of the high-fat high-fructose diet and the partial prevention induced by the *Opuntia stricta* var. *dillenii* peel extract, the effects of dietary treatments on the metabolic pathways involved in adipose tissue triglyceride metabolism were assessed. Concerning epididymal adipose tissue, both FAS protein expression and activity were significantly reduced in rats fed the high-fat high-fructose diet (HFHF group), when compared to the controls, suggesting an inhibition of de novo lipogenesis, which provides fatty acids required for triglyceride synthesis [41]. This effect can represent a compensatory response with the aim of limiting excessive fat accumulation in the tissue under this feeding pattern. In addition to this source of fatty acids, this lipid species can also originate from the bloodstream. Circulating free fatty acids are taken up by specific transporters (CD36, FATP1), while fatty acids integrated in circulating triglycerides (chylomicrons and VLDL) are taken up by LPL, an enzyme located on the vascular endothelial surface. In epididymal adipose tissue, the obesogenic diet increased CD36 expression, suggesting enhanced fatty acid availability for triglyceride synthesis, without changes in LPL levels. As aquaglyceroporins play a crucial role in glycerol transport, protein expression of AQP7, responsible for glycerol release, and AQP9, responsible for glycerol uptake, were measured [42], but no changes were induced by high-fat high-fructose diet feeding. It is important to emphasize that triglyceride synthesis needs, in addition to fatty acids, glycerol-3P (G3P), given that in adipose tissue the activity of glycerol kinase (GK), is negligible [43]. Because the source of G3P in adipose tissue is glucose metabolism, we also measured protein expression of glucose transporter GLUT4, which is stored in intracellular vesicles under basal conditions and translocated to the membrane in response to insulin, to facilitate glucose uptake into the adipocyte [44]. The induced significant increase in this transporter indicates enhanced glucose uptake by the cells. Regarding triglyceride assembly, it has been reported that DGAT1 and DGAT2 catalyse the same reaction and they can largely compensate for each other for triglyceride storage. However, whereas DGAT1 uniquely has an important role in protecting the endoplasmic reticulum from the lipotoxic effects of high-fat diets, DGAT2 is the predominant enzyme for triglyceride storage in adipose tissue in the same feeding condition [45]. Therefore, DGAT2 analysis was prioritised over DGAT1. Under our experimental conditions, a boost in DGAT2 was observed, suggesting increased triglyceride formation.

Lastly, since lipolytic enzymes regulate the mobilization of stored fats for energy production, they were also studied. Overall, the markers assessed suggest that lipolysis was not affected by the obesogenic diet. Altogether, the present results show that under high-fat high-fructose feeding, fattening was induced by increasing substrate availability (fatty acid and glycerol-3P) and the triglyceride assembly processes involved in triglyceride synthesis.

Concerning the effects of the *Opuntia stricta* var. *dillenii* extract on epididymal adipose tissue, the decrease observed in FAS protein expression and activity in rats from HFHF group was totally prevented by both doses, most likely as a result of reduced adipose tissue mass, the mentioned compensatory mechanism that took place in HFHF group was no longer needed. Moreover, both doses induced a strong reduction in FATP1 protein expression, and the low dose prevented the increase in GLUT4 observed in rats fed the obesogenic diet. Surprisingly, the high dose induced a strong increase in the expression of CD36. This transporter has been shown to release long-chain fatty acids from adipocytes under lipolytic conditions, acting in the opposite way to its function under insulin signaling [46]. Given that the expression of both lipases was increased in the ODH group, an increase in fatty acid release, potentially linked to the up-regulation of CD36, can be proposed. Finally, both doses prevented the increase in DGAT2 protein expression induced by high-fat high-fructose feeding. Collectively, these results indicate that, in epididymal tissue, the *Opuntia* extract prevented the rise in the availability of substrates needed for triglyceride synthesis induced by HFHF feeding, as well as triglyceride assembly. In the case of the high dose, the involvement of increased lipolysis is also proposed.

As far as subcutaneous depot is concerned, the fattening effect induced by high-fat high-fructose feeding in this adipose depot was associated with increased fatty acid availability, mediated by FATP1 and LPL, without changes in glucose uptake and thus, in G3P availability. Moreover, decreased lipolysis, as shown by decreased ATGL protein expression could have also contributed to the increase in adipose tissue size. In this tissue, as in the epididymal depot, the decreased size induced by the *Opuntia* extract did not appear to be related to changes in de novo lipogenesis. Concerning substrate availability, the reduction in FATP1, that reached statistical significance in ODH group, indicates a decrease in fatty acids disposal for triglyceride synthesis. In addition, unlike the epididymal fat pad, the findings in subcutaneous fat suggest that glucose availability was not altered. Finally, similarly to that found in epididymal adipose tissue, increased protein expression of ATGL and activation of HSL indicate increases in lipid mobilization. The effect on the lipases was accompanied by a higher expression of AQP7, the protein involved in glycerol output from adipocytes. These results show that the number of metabolic pathways affected by the *Opuntia* extract in subcutaneous depot was lower than in epididymal adipose tissue, thus supporting the fact that only the latter was significantly reduced.

**Figure 11:**
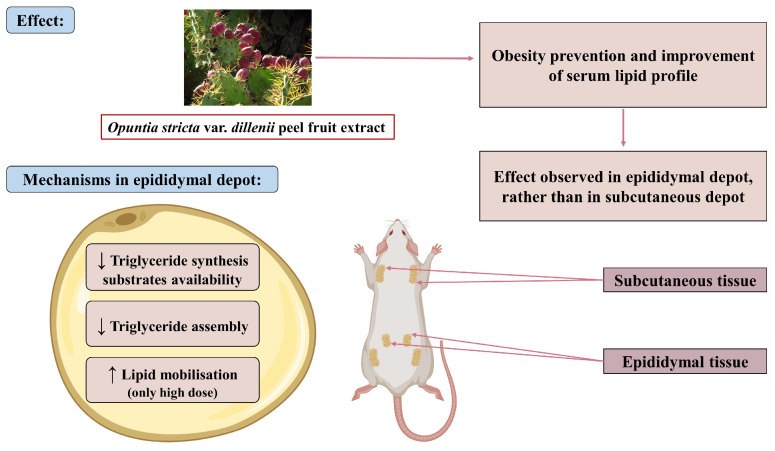
Schematic representation of the effects and mechanisms of action of *Opuntia stricta* var. *dillenii* extract in rats fed an obesogenic diet. ↑: increase; ↓: decrease (Created using BioRender https://BioRender.com).

To provide a more general overview, a figure has been prepared summarising all the results and the proposed mechanisms underlying the observed effects (Figure 11).

## 5. Conclusions

In conclusion, under the present experimental conditions, the supplementation with *Opuntia stricta* var. *dillenii* extract obtained from the peel fruit exerts a preventive effect on obesity development by partially attenuating HFHF-induced fat accumulation. This effect was observed in visceral, but not in subcutaneous adipose tissue. The obesity prevention was accompanied by the improvement of serum lipid profile. The mechanisms underlying the extract anti-obesity effect, which was analysed in epididymal adipose tissue, involve preventing the rise in the availability of triglyceride synthesis substrates induced by high-fat high-fructose feeding, the inhibition of triglyceride assembly, and in the case of the high dose, increased lipolysis. Thus, according to these results, the peel wastes of *Opuntia stricta* var. *dillenii* fruit represent a potential natural source of bioactive compounds with preventive effects against diet-induced obesity.

## Figures and Tables

**Table 1:** Quantification of the main betalains and phenolic compounds present in *Opuntia stricta* var. *dillenii* peel extract by HPLC-DAD-MS.

Compound	Content (µg of Compound/g Dry Weight)
Piscidic acid	19,269 ± 382
Betanin	5160 ± 88
Isobetanin	3038 ± 95
2′-O-apiosyl-4-O-phyllocactin	1372 ± 102
Isorhamnetin glucoxyl-rhamnosyl-pentoside (IG2)	989 ± 21
Neobetanin	429 ± 11
Quercetin glycoside 2 (QG2)	245 ± 9
Quercetin-3-O-rhamnosyl-rutinoside (QG3)	202 ± 6
5″-O-E-sinapoyl-2-apiosyl-phyllocactin	183 ± 37

**Table 2:** Nutrient composition of the experimental diets.

Composition by Energy (%)	Standard Diet	High-Fat High-Fructose Diet (HFHF)
Protein	20.3	20.0
Carbohydrate	63.9	40.0
Fructose	-	10.0
Lipid	15.8	40.0
Total	100.0	20.0
Energy (kcal/g)	3.9	4.5

**Table 3:** Food and energy intake, final body weight, weight gain, adipose pad weight and white adipose tissue index in rats fed a standard diet (C) and a high-fat high-fructose diet alone (HFHF) or supplemented with *Opuntia stricta* var. *dillenii* peel extract at a dose of 25 mg/kg weight/day (ODL) or 100 mg/kg weight/day (ODH).

	C	HFHF	ODL	ODH	ANOVA
Food intake (g/day)	19.3 ± 0.3 ^a^	20.4 ± 0.4 ^a^	20.1 ± 0.6 ^a^	19.5 ± 0.2 ^a^	NS
Energy intake (kcal/day)	76.6 ± 1.1 ^b^	91.7 ± 1.8 ^a^	90.1 ± 2.5 ^a^	87.6 ± 0.7 ^a^	*p* < 0.05
Body weight (g)	425.0 ± 7.4 ^b^	473.5 ± 10.6 ^a^	459.0 ± 12.0 ^a^	460.0 ± 8.0 ^a^	*p* < 0.05
Body weight gain (g)	232.3 ± 9.9 ^b^	281.4 ± 12.3 ^a^	267.1 ± 13.6 ^ab^	266.1 ± 10.8 ^a^	*p* < 0.05
Total white AT weight (g)	40.7 ± 2.2 ^c^	61.0 ± 2.3 ^a^	50.5 ± 4.0 ^b^	53.0 ± 3.3 ^b^	*p* < 0.05
Subcutaneous AT weight (g)	13.4 ± 0.7 ^b^	19.0 ± 0.9 ^a^	16.5 ± 1.2 ^a^	16.6 ± 1.3 ^a^	*p* < 0.05
Visceral AT weight (g)	30.8 ± 2.8 ^b^	43.7 ± 1.4 ^a^	36.4 ± 3.2 ^b^	36.4 ± 2.3 ^b^	*p* < 0.05
Epidydimal AT weight (g)	10.0 ± 0.6 ^c^	16.5 ± 0.8 ^a^	14.6 ± 1.4 ^ab^	14.3 ± 1.0 ^b^	*p* < 0.05
Perirenal AT weight (g)	12.8 ± 0.9 ^b^	19.1 ± 0.9 ^a^	17.8 ± 1.1 ^b^	17.4 ± 1.2 ^a^	*p* < 0.05
Mesenteric AT weight (g)	4.5 ± 0.4 ^c^	6.3 ± 0.4 ^a^	5.6 ± 0.5 ^ab^	5.4 ± 0.2 ^b^	*p* < 0.05
White adipose index (%)	9.6 ± 0.5 ^c^	12.7 ± 0.3 ^a^	11.1 ± 0.7 ^bc^	11.5 ± 0.6 ^ab^	*p* < 0.05

White adipose index was calculated as the percentage of total white adipose tissue relative to body weight. AT: adipose tissue; d: day. The values are presented as mean ± SEM. Values within the same row displaying different letters differ significantly (*p* < 0.05). NS: not significant.

**Table 4:** Serum biochemical variables.

	C	HFHF	ODL	ODH	ANOVA
Triglycerides (mg/dL)	100.4 ± 2.1 ^a^	93.7 ± 5.1 ^ab^	92.3 ± 2.4 ^b^	46.7 ± 9.9 ^c^	*p* < 0.05
Total Cholesterol (mg/dL)	63.6 ± 5.4 ^c^	123.0 ± 4.3 ^a^	106.4 ± 6.4 ^b^	119.7 ± 2.5 ^ab^	*p* < 0.05
HDL Cholesterol (mg/dL)	13.5 ± 0.7 ^b^	13.1 ± 0.4 ^b^	12.5 ± 1.5 ^b^	20.3 ± 1.8 ^a^	*p* < 0.05
Non-HDL Cholesterol (mg/dL)	50.1 ± 5.5 ^c^	109.4 ± 4.6 ^a^	92.4 ± 5.8 ^b^	99.6 ± 2.5 ^ab^	*p* < 0.05
NEFA (mmol/L)	0.9 ± 0.1 ^a^	0.8 ± 0.1 ^a^	0.7 ± 0.1 ^a^	0.7 ± 0.1 ^a^	NS
Leptin (ng/mL)	2.0 ± 0.5 ^a^	1.8 ± 0.3 ^a^	1.5 ± 0.3 ^a^	1.5 ± 0.2 ^a^	NS
Adiponectin (ng/mL)	4.7 ± 0.1 ^a^	4.7 ± 0.1 ^a^	4.7 ± 0.1 ^a^	4.7 ± 0.2 ^a^	NS

HDL: high-density lipoprotein; NEFA: non-esterified fatty acids. The values are presented as mean ± SEM. Values within the same row displaying different letters differ significantly (*p* < 0.05). NS: not significant.

## Data Availability

The original contributions presented in this study are included in the article. Further inquiries can be directed to the corresponding author.
